# Abnormal yawning in stroke patients: the role of brain thermoregulation

**DOI:** 10.3389/fnins.2014.00300

**Published:** 2014-09-17

**Authors:** Andrew C. Gallup

**Affiliations:** Psychology Department, State University of New York at OneontaOneonta, NY, USA

**Keywords:** yawning, stroke, brain temperature, thermoregulation, excessive yawning

Krestel et al. (2013) recently investigated the potential contributing factors associated with abnormal yawning (defined as ≥3 yawns/15 min) in 10 patients with acute anterior circulation stroke. Though frequent yawning had previously been observed in stroke patients (Cattaneo et al., 2006; Singer et al., 2007), this study attempted to assess the influence of specific physiologic and lesion topographic variables contributing to this association. All patient parameters were taken within 1 h after admission and emergency nurses recorded a single axillary body temperature with a digital thermometer (Krestel, personal communication). Using MRI lesion maps, and reportedly finding no associations between various physiologic measures, including blood oxygen saturation, glucose, body temperature, blood pressure, and heart rate, the authors concluded that ischemic lesions in the posterior insula and caudate nucleus might facilitate high frequency yawning in stroke patients. While this report improves our neurological understanding regarding the association between frequent yawning in stroke patients, limitations in the analysis and interplay of temperature need to be addressed.

Yawning is characterized by a powerful gaping of the jaw with inspiration, a brief period of peak muscle contraction, and a passive closure of the jaw with shorter expiration (Barbizet, 1958). The localized circulatory changes resulting from this action pattern have led researchers to hypothesize that yawns may function to cool the brain (Gallup and Gallup, 2007). For example, yawns produce increases in blood flow to the skull and enhanced venous return (Bhangoo, 1974), while the deep inhalation during yawning can modify the temperature of venous blood draining from the nasal and oral orifices into the cavernous sinus, which surrounds the internal carotid artery (Zenker and Kubik, 1996). Together, these processes act like a radiator removing hyperthermic blood from brain while introducing cooler arterial blood to the brain. Moreover, the flexing of the musculature during yawning may encourage the evaporation of the sinus mucosa (see Gallup and Hack, 2011). Research supporting the brain cooling hypothesis has accumulated over the past 5 years, including evidence for predicted brain and body temperature fluctuations surrounding yawning events, indirect manipulations of brain temperature causing a reduction in yawn frequency, and an altered expression of yawning which appears to be driven by ambient temperature manipulation/variation (reviewed by Gallup and Eldakar, 2013). For example, by directly monitoring continuous changes in prelimbic cortex temperature of rats it was shown that yawning is preceded by intermittent and rapid increases in brain temperature (i.e., ~0.1°C/min), and that following the completion of a yawn the slope of the temperature change reverses and quickly returns to baseline (Shoup-Knox et al., 2010).

While the breadth of physiologic measures taken by Krestel et al. (2013) is laudable, a single measure of axillary temperature taken long after the onset of the yawning episode is inadequate for assessing this relationship. Since distinct brain temperature changes in rats occur before and after single yawns on a rather short temporal scale; i.e., 60–90 s (Shoup-Knox et al., 2010), and isolated bouts of excessive yawning in humans have been shown to reduce skull temperature by as much as 0.4°C (Gallup and Gallup, 2010), it remains possible that the pathological yawning experienced by these patients was accompanied by recurrent changes in temperature that were never recorded. Furthermore, temperature measures taken from the skull (e.g., oral, tympanic, forehead) would be more informative since the motor pattern of yawning and the associated circulatory changes are localized to this area. That said, even these measurements could miss important temperature fluctuations confined to particular brain regions.

The use of MRI lesion maps to establish a relationship between ischemic lesions in the posterior insula and caudate nucleus and the duration of abnormal yawning is certainly of great importance (Krestel et al., 2013). At this point, however, it is premature to declare that there was “no evidence of other potential causes” related to abnormal yawning in these patients. To the contrary, frequent or abnormal yawning in stroke patients may be a consequence of thermoregulatory dysfunction associated with the brain injury (Gallup and Gallup, 2008). Given the close temporal association between yawning and changes in brain/skull temperature, future research monitoring patients with abnormal or excessive yawning bouts should take continuous temperature measures from areas proximate to the cranium in order to properly assess this relationship.

## Conflict of interest statement

The author declares that the research was conducted in the absence of any commercial or financial relationships that could be construed as a potential conflict of interest.
